# Hypoxic Preconditioning: An Underestimated Endogenous Protective Strategy?

**DOI:** 10.3390/biom16070976

**Published:** 2026-07-02

**Authors:** Jinqiao Liu, Changhong Ren, Tariq Kermalli, Yuchuan Ding, Xunming Ji, Guowei Lu, Sijie Li

**Affiliations:** 1 Department of Neurology, Xuanwu Hospital, Capital Medical University, Beijing 100053, China; liujqiao@outlook.com (J.L.); jixm@ccmu.edu.cn (X.J.); 2 Beijing Key Laboratory of Hypoxic Conditioning Translational Medicine, Xuanwu Hospital, Capital Medical University, Beijing 100053, China; rench@xwhosp.org (C.R.); gwlu@ccmu.edu.cn (G.L.); 3 Department of Neurosurgery, Wayne State University School of Medicine, Detroit, MI 48201, USA; hp8235@wayne.edu (T.K.); yding@med.wayne.edu (Y.D.); 4 Neuro Cardio Vascular Diseases Center, Xuanwu Hospital, Capital Medical University, Beijing 100053, China; 5 Beijing Institute of Brain Disorders, Capital Medical University, Beijing 100069, China; 6 Clinical Center for Combined Heart and Brain Disease, Capital Medical University, Beijing 100069, China

**Keywords:** hypoxia, hypoxic preconditioning, acquired tolerance, brain plasticity, intrinsic cytoprotection, conditioning medicine

## Abstract

Hypoxic Preconditioning (HPC) describes the phenomenon whereby brief, non-lethal hypoxic episodes enhance cellular resilience to subsequent severe hypoxia. Initially recognized in altitude studies, HPC has since been linked to broad protective effects, particularly in the protection of ischemia/hypoxia. This article highlights the biological rationale, potential mechanisms and applications, while addressing the challenges of clinical translation. As a form of endogenous adaptation, HPC offers a promising paradigm for preventive and therapeutic strategies across various diseases.

## 1. Introduction

Hypoxia, or the condition of low oxygen levels, poses a significant risk to humans due to our limited tolerance of such environments. However, it is important to note that many cells, both in nature and within the human body, frequently encounter hypoxic conditions. The ability of these cells to withstand hypoxia is primarily determined by their intrinsic adaptive mechanisms, which allow them to survive and function under oxygen-deprived conditions [1,2].

Hypoxia should therefore not be regarded only as a harmful pathological state. Instead, it represents a complex biological signal whose effects depend on its intensity, duration, frequency, and cellular context [3]. Severe, prolonged, or uncontrolled hypoxia may result in metabolic collapse, oxidative stress, inflammation, irreversible tissue injury, and even death. In contrast, mild, short-term, and well-controlled hypoxic exposure may activate endogenous protective pathways and improve the ability of cells and organs to resist subsequent injury [4,5]. Researchers have uncovered various benefits associated with hypoxia, which led us to introduce the concept of Hypoxic Preconditioning (HPC) [6]. This concept proposes that after experiencing brief, non-lethal episodes of repeated hypoxia, the body’s cells develop increased resilience to prolonged, potentially fatal hypoxic conditions [7,8].

The dual nature of hypoxia—being both a potential threat and a possible therapeutic stimulus—has fundamentally reshaped our understanding of oxygen biology in recent decades [9]. Traditional medical paradigms have long emphasized the restoration of normoxia as the primary therapeutic goal, often overlooking the adaptive potential embedded in controlled hypoxic exposure. Yet emerging evidence suggests that the body’s response to oxygen deprivation is far more nuanced than previously appreciated, involving sophisticated sensing mechanisms, transcriptional reprogramming, and metabolic flexibility that can be harnessed for therapeutic benefit [10]. This paradigm shift invites us to reconsider hypoxia not merely as a pathological condition to be avoided, but as a biological signal that can be precisely modulated to enhance cellular resilience.

In this sense, HPC can be understood as a typical form of acquired tolerance. It is not simply a passive response to oxygen deprivation, but an active adaptive process involving metabolic remodeling, changes in gene expression, regulation of ion homeostasis, inhibition of excessive oxidative injury, and activation of anti-apoptotic and anti-inflammatory mechanisms [11]. The core idea of HPC may be summarized as “combating hypoxia with hypoxia.”

Building on earlier studies, research into HPC has paved the way for numerous practical applications. At present, HPC-related strategies are being explored in the prevention and treatment of ischemic stroke, myocardial ischemia–reperfusion injury, high-altitude disease, neurodegenerative disorders, inflammatory diseases, immune dysfunction, and even cancer-related conditions. In this review, we highlight the origin concept of HPC at a time when multiple ways of hypoxic preconditioning are emerging, and examine its latest advancements. However, many previous reviews have focused primarily on specific mechanisms, individual organs, or particular disease settings. In contrast, the present review aims to provide a broader integrative perspective by tracing HPC from the biological nature of hypoxia and the universality of adaptive responses to its molecular basis, systemic protective effects, and translational potential. By linking the historical origin, conceptual development, and physiological foundation of HPC, this review highlights HPC not only as a mechanistic phenomenon, but also as an endogenous adaptive strategy rooted in hypoxia tolerance biology.

## 2. Multiplicity of Hypoxia

Oxygen is essential for the survival of humans and most organisms on Earth, playing a critical role in metabolic processes and energy production [12]. It is vital for sustaining life and supporting human physiological activities. Oxygen is essential for the prosperity of life, yet low-oxygen environments are widespread in nature and under special conditions [13,14,15]. While humans and most animals normally thrive in oxygen-rich environments at the Earth’s surface, hypoxic or anoxic conditions occur in settings such as high-altitude regions, deep-sea habitats, space, and underwater environments, as well as in unusual gaseous or physical conditions [16,17]. According to its origin, hypoxia can generally be divided into environmental hypoxia, physiological hypoxia, and pathological hypoxia [18]. Environmental hypoxia is typically caused by a decrease in the partial pressure of inspired oxygen, as occurs at high altitude. Physiological hypoxia refers to the relatively low oxygen tension that naturally exists in many tissues and organs under normal conditions [19]. Pathological hypoxia results from abnormalities in oxygen uptake, transport, delivery, or utilization [2]. These different types of hypoxia are not completely independent; instead, they often interact with each other in clinical and experimental settings [3].

As plateaus are a common unusual hypoxic environment, altitude ranges are usually divided into four distinct regions: 1. A zone from ground level to an altitude of 2000 m where labor can be carried out without the need for compensatory functions; 2. A fully compensatory zone from 2000 m to 4000 m; 3. An incomplete compensatory zone between 4000 and 7000 m where the unaccustomed population experiences various physiological disturbances. The 4000 m mark is regarded as a safety threshold, as surpassing this altitude can significantly impact both physical and mental functions in humans [20]. While these effects are detrimental, they are generally reversible upon descent; 4. At an altitude above 7000 m, the equivalent O_2_ concentration falls below 8.5%, potentially resulting in life-threatening conditions such as loss of consciousness and muscle spasms [21,22,23,24,25]. The approximate relationship among altitude, ambient oxygen partial pressure, equivalent O_2_ concentration, and representative physiological effects is summarized in Table 1. The classification of altitude zones indicates that hypoxic stress has a clear dose-dependent feature [26]. At relatively low altitudes, the body can usually compensate through increased ventilation, enhanced cardiac output, and changes in oxygen transport. However, when the degree of hypoxia exceeds the compensatory capacity of the organism, systemic dysfunction and cellular injury may occur [16]. This dose–response relationship is also important for understanding why controlled hypoxia can be protective, whereas excessive hypoxia is harmful.

Natural altitude hypoxia initially manifests as a harmful stressor, which has prompted extensive investigation into the biological effects of altered oxygen availability. These studies have also highlighted that hypoxic consequences may arise in certain hyperoxic environments [27]. For example, prolonged breathing of 100% oxygen, particularly under hyperbaric conditions, can increase the risk of absorption atelectasis [28]. Under high inspired oxygen fractions, alveolar nitrogen is washed out and replaced by oxygen. Because nitrogen is an inert gas with low blood solubility and normally helps maintain alveolar patency, its removal renders the alveoli more susceptible to collapse [29]. In lung regions with reduced ventilation/perfusion ratios, oxygen is absorbed into the blood more rapidly than it can be replenished by ventilation, leading to progressive alveolar shrinkage until surface-tension forces trigger collapse. This collapse can impair pulmonary gas exchange and contribute to secondary hypoxemia; accordingly, intermittent air breaks are often used clinically to reduce this risk [30].

However, low oxygen levels are actually common in the human body, as the normal physiological environment within the body itself constitutes a type of low-oxygen condition [31,32]. For example, oxygen tension in the heart, liver, and kidneys typically ranges from 4% to 14% [33,34], whereas in brain tissue it is generally lower, ranging from approximately 0.5% to 7% depending on the region and local microenvironment [35]. Such relatively low physiological oxygen levels are not necessarily pathological. On the contrary, it is essential for maintaining normal tissue homeostasis. For instance, physiological hypoxia participates in embryonic development, angiogenesis, stem cell maintenance, bone marrow function, and immune-cell regulation. Many cells have evolved oxygen-sensing systems that allow them to adjust their behavior according to local oxygen availability.

Hypoxia is also a common pathological process and mechanism in various clinical diseases and plays a fundamental role in the body’s eventual death [36,37]. Any malfunction in the supply of environmental oxygen, along with the uptake, transportation, or utilization of oxygen in the body, could ultimately lead to hypoxia. Various types of hypoxia such as hematological, circulatory ischemic, and tissue hypoxia are commonly seen in clinical practice. Metabolic, functional and structural disorders may occur in the body’s organs and tissues, especially in the nervous system when the oxygen supply to tissue cells is insufficient, or oxygen utilization is impaired [38,39]. The hypoxic metabolic pathway is closely related to the occurrence and development of various diseases such as cardiovascular disease and some tumors [40].

Beyond these classical categorizations, contemporary research has revealed that hypoxia operates across multiple temporal and spatial scales within biological systems. At the subcellular level, mitochondrial oxygen gradients can vary dramatically even within a single cell, creating microenvironments that influence local signaling cascades. At the tissue level, oxygen gradients are essential for processes such as stem cell niche maintenance, where the hypoxic environment of the bone marrow stem cell niche preserves stemness and prevents premature differentiation. At the organismal level, circadian fluctuations in tissue oxygenation reflect the dynamic interplay between metabolic demand and supply. This multi-scale complexity underscores why a one-size-fits-all approach to hypoxia—whether viewing it as universally harmful or universally beneficial—fails to capture the biological reality.

Therefore, hypoxia is a multifaceted biological phenomenon. It can be an environmental challenge, a physiological condition, a pathological mechanism, and a therapeutic stimulus. This multiplicity provides the basis for the development of HPC, because the body’s response to hypoxia is not fixed but depends on the pattern of exposure and the adaptive capacity of cells and tissues.

## 3. Universality of Adaptation

From sea level, where atmospheric pressure and oxygen concentration are optimal, to the high plateaus with sparse air, every corner of the Earth’s surface showcases life’s remarkable persistence, continuous adaptation, and evolution. While humans and animals at sea level typically require an oxygen concentration of about 20% to thrive, some species have evolved to survive in extreme environments where they can withstand and adapt to the severe conditions [41]. For example, some mammals like rock sheep, brown bears, and yaks can move freely at an altitude of 6000–8000 m. Similarly, Andean highlanders living at high altitude in the Andes, including regions of Peru, Bolivia, northern Chile, and northwestern Argentina, provide an important model for studying human adaptation to chronic hypobaric hypoxia [42]. This adaptive phenotype should be distinguished from chronic mountain sickness, a pathological condition characterized by excessive erythrocytosis and sometimes cyanosis [43]. Turtles, as facultative hypoxia-tolerant animals, can tolerate hypoxia for extended periods of time by downregulating their energy demand and upregulating their ATP generation efficiency; ATP is maximally obtained from every mol of O_2_ under low oxygen conditions, while ATP is maximally gained from every mol of H^+^ through anaerobic metabolic pathways under anaerobic conditions [44]; some aquatic vertebrates can even manage environments with severe hypoxia given previous exposure to hypoxia and adenosine-dependent mechanisms [45]. Also, some prokaryotic cells such as Escherichia coli have several different respiratory chains and can survive in anaerobic environments using different electron acceptors and terminal oxidoreductases [46]. Additionally, researchers have identified a natural adaptive phenomenon whereby re-exposure to a high-altitude environment leads to faster acclimatization than the initial exposure, potentially involving endogenous adenosine-dependent mechanisms [47]. Similarly, spearfishermen have been reported to exhibit enhanced cardiovascular efficiency, possibly through related physiological mechanisms [48].

These various species navigating hypoxic environments illustrate the effects of adaptation, which is a common phenomenon and even a driver of evolution in the biological world. Adaptation can be understood in two ways: firstly, the structural specialization gradually made from adaptation caters for specific functions: for example, the wing structure of birds is suitable for flight, and the structure of human eyes is ideal for sensing objects and images [49]. Secondly, the structure and function of organisms ensure their survival and continuation in dynamic environmental conditions, with an example being the body shape of fish and their ability to breathe through gills, which enable them to live in water.

In addition to long-term evolutionary adaptation, organisms also possess short-term adaptive responses [3]. These responses may occur within minutes, hours, or days and involve changes in ventilation, circulation, metabolism, enzyme activity, neurotransmitter release, ion-channel function, and gene transcription. HPC belongs to this category of short-term acquired adaptation [4]. Unlike evolutionary adaptation, HPC does not require genetic selection across generations. Instead, it relies on the activation of protective mechanisms within an individual organism after controlled exposure to hypoxia.

The remarkable diversity of adaptive strategies across species offers valuable insights for translational medicine. By studying how hypoxia-tolerant organisms have evolved to thrive in oxygen-limited environments, researchers can identify conserved molecular pathways that may be therapeutically targeted in humans. For instance, the metabolic suppression observed in hibernating mammals and diving marine animals shares common features with the protective state induced by HPC, suggesting that certain evolutionary adaptations can be transiently reactivated through controlled hypoxic exposure. This cross-species perspective bridges the gap between evolutionary biology and clinical medicine, providing a rich source of inspiration for novel therapeutic approaches.

Although adaptation has significant potential, it is not unlimited since it is characterized by a dose–response relationship [26]. Furthermore, the effects of hypoxia are frequently complex and multifaceted [50]. High levels of stress or injury can trigger harmful reactions in the body, while low levels can stimulate protective strategies. The combination of hypoxia and adaptation also follows this rule. We have discussed the detrimental effects of excessive hypoxia, including loss of consciousness, cognitive dysfunction, and even central nervous system diseases. However, the effects of moderate hypoxia exposure on organisms are quite different [51]. Hypoxia is associated with an array of severe environments that are commonly endured by many species, and one of the strategies to combat hypoxia is adaptation. The activity and survival of animals in a hypoxic environment depend on the adaptive changes in tissue cells and molecular genes in the body, one type of which, hypoxic preconditioning, is an essential biological strategy that combats hypoxia with hypoxia [6,52,53].

## 4. Combat Hypoxia with Hypoxia

The concept of mithridatism, named after King Mithridates VI of Pontus, refers to the practice of administering small, non-lethal doses of a harmful substance to gradually develop resistance to its toxic effects [54]. A similar idea can also be found in traditional Chinese medicine. The Yellow Emperor’s Inner Canon, one of the earliest extant medical classics in China, contains indirect discussions related to this principle, while Shennong Bencao Jing, the earliest known Chinese pharmacological monograph, provides a more explicit description of using toxic agents in a controlled manner for therapeutic purposes. Conceptually, this reflects the same general logic as HPC: a mild, controlled, and non-lethal stress stimulus can activate endogenous protective mechanisms and thereby increase tolerance to a subsequent, more severe insult. Although hypoxia differs fundamentally from poisoning, HPC is based on a comparable adaptive principle, namely “using hypoxia to resist hypoxia.” This concept has gradually become an important preventive and therapeutic strategy, particularly in hypoxia- and ischemia-related diseases, and has contributed to the development of HPC as a recognized biomedical concept [55,56,57].

### 4.1. Hypoxic Preconditioning, an Acquired Tolerance: History, Definition and Its Implementation

#### 4.1.1. Confirmation of Hypoxic Effects

Under hypoxic conditions, the positive response of the body’s organ system to maintain homeostasis is known as hypoxia tolerance response. It is usually characterized by changes in lung ventilation and cardiovascular function [58]. However, the enhancement of organ system functional activity is not sufficient to explain the tolerance of humans and animals to hypoxia. In 1927, Haldane identified the phenomenon as “physicochemical brain”. Many early studies also confirmed the phenomenon [59]. Populations tolerant of low oxygen, such as high-altitude indigenous residents, do not exhibit enhanced organ system activity [42]. In a hypoxic environment or with cardiovascular diseases, even if organ system activity is enhanced, it does not help the body combat hypoxia. Additionally, individuals at lower developmental stages within the same species exhibit higher tolerance to low oxygen levels, despite their organ systems showing a less pronounced response [60]. The respiratory and circulatory responses in adult animals are initially strong after tracheal clamping; however, blood pressure drops sharply to zero in less than five minutes. In contrast, although infants and young animals show no significant organ system responses by the third day after birth, their blood pressure takes 17 min to decrease to 60% of normal levels [6]. Experimental studies have consistently shown that fetal and neonatal mammals display greater tolerance to hypoxia and ischemia than adults, a property often attributed to lower metabolic demand, greater metabolic flexibility, and reduced excitotoxic vulnerability [61]. These examples are difficult to explain by traditional knowledge of organ system adaptation reactions, and are even considered “biological phenomena that could not be explained from a physicochemical perspective.”

In 1963, our team understood this difficult-to-explain phenomenon as an ‘acquired tolerance’ occurring at the cellular level in the body, reported the tissue mechanisms underlying adaptation to hypoxia, proposed the concept of hypoxic preconditioning, and emphasized the role of tissue cells and molecular genes in this protective process [6]. In 1986, Murry’s team reported that the heart’s ability to tolerate ischemic damage was significantly enhanced after repeated ischemia, and proposed the concept of ischemic preconditioning (IPC) [62]. Since the essence of IPC is nothing more than the adaptation of the body’s tissue cells to low oxygen, we can unify the two concepts as follows: after a short period of non-lethal repeated hypoxia/ischemia in advance, the body’s tissue cells obtain high tolerance to the subsequent long-term lethal hypoxia/ischemia injury.

**Figure 1:**
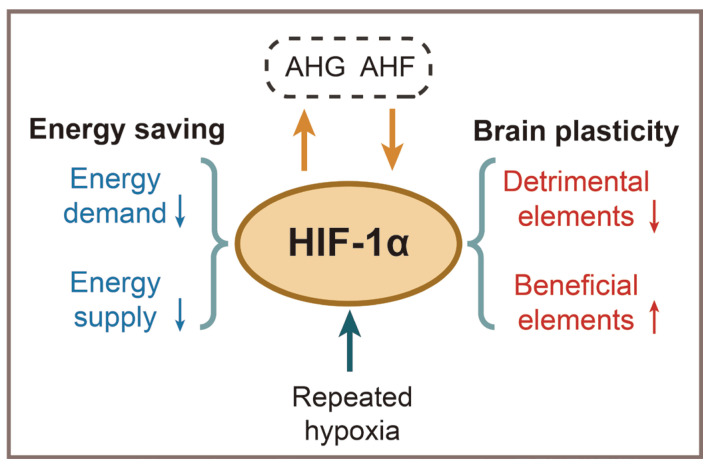
Hypoxia-induced increases in HIF-1-based energy saving, brain plasticity, and upregulation of anti-hypoxia-related genes. **Abbreviations:** AHG, anti-hypoxia related genes; AHF, anti-hypoxia factor.

Following confirmation of this concept, many scientists have conducted numerous studies on this topic [63]. With the advancement of research in HPC, its positive effects on multiple organs have been identified. To gain a better understanding, we employed a whole-body HPC model to induce self-hypoxia in animals. In this model, we observed that the second, third, fourth, and fifth hypoxia tolerance limits of animals exposed to repeated hypoxia have a tolerance time 2, 4, 6, and 8 times longer than the first hypoxia tolerance limit, respectively [64]. The survival time of animals exposed to 4 or 5 repeated episodes of hypoxia in a low-pressure chamber and under the action of potassium cyanide is 10 and 4 times longer than that of normal control animals respectively. Some studies targeting it were also carried out, which have found that normal animals injected with brain homogenate extract, when exposed to 4 or 5 repeated hypoxic conditions into the abdominal cavity, have a survival time of 1.8 or 2 times longer in a low-pressure chamber compared to animals receiving physiological saline or normal animal brain homogenate extract respectively [65]. Others discovered that brain homogenate extract from animals subjected to repetitive hypoxia has significant protective effects on the cultivation of PC12 cells, the preparation of synaptic bodies in the cerebral cortex and resistance to hypoxic damage [66]. The animals’ prolonged survival, primarily due to the prevention of hypoxic death caused by the failure of the central nervous system, indicates its remarkable neuroprotective effects. So, researchers highlighting the neuroprotective mechanisms involving metabolic and molecular changes, utilized Hypoxia-inducible factor 1-alpha (HIF-1α) activation (Figure 1) and found that with an increasing number of repeated hypoxic exposures, the oxygen consumption rate in animals decreased exponentially [67]. In addition to HIF-mediated oxygen sensing, adenosine signaling represents another rapid and sensitive pathway involved in the response to hypoxia. During hypoxia, ischemia, and other forms of energy depletion, ATP breakdown promotes adenosine accumulation, which regulates tissue adaptation and recovery through A1- and A2-receptor-mediated signaling [68]. This mechanism has been extensively studied in the heart, where adenosine contributes to hypoxic preconditioning by reducing cardiac workload and promoting vasodilation and tissue oxygenation. Repeated hypoxic preconditioning induces coordinated molecular changes characterized by reduced detrimental factors and enhanced protective factors that together improve brain tolerance to hypoxia and ischemia (Table 2). It should be noted that these molecular responses have been reported across different repeated hypoxic preconditioning paradigms, including whole-body autohypoxia, low-pressure hypoxia, and intermittent hypoxia exposure, and therefore represent convergent patterns rather than changes observed under one uniform protocol.

Beyond the early foundational work from our group, independent studies have increasingly supported and refined this concept. For instance, controlled hypoxia has been shown to induce a hibernation-like state in non-hibernating mice, characterized by markedly reduced oxygen consumption and carbon dioxide production, lowered body temperature, and significant neuroprotection against focal cerebral ischemia, suggesting that hypoxia-induced metabolic suppression itself may contribute to brain protection [69]. More recently, Liu et al. demonstrated that intermittent hypoxia preconditioning (13% O_2_, 5 min hypoxia/5 min normoxia, 10 cycles/day for 2 weeks) protects against ischemic brain injury in mice via a PF4-dependent paracrine mechanism. In that study, plasma from intermittent hypoxia-preconditioned mice transferred neuroprotection to recipient animals, while PF4 immunodepletion abolished this effect and recombinant PF4 recapitulated the benefit across sex and age groups [70]. These findings not only independently corroborate the neuroprotective effects of hypoxic preconditioning, but also extend earlier observations by identifying a specific circulating mediator that links systemic hypoxic preconditioning to cerebrovascular protection. To date, organ protection induced by HPC has been confirmed [57], but the underlying mechanisms remain largely unclear. So far, it is only known that preconditioning activates certain genes that may contribute to enhanced acquired tolerance.

The translation of these conceptual frameworks into clinical practice represents one of the most exciting frontiers in modern medicine. Bridging the gap between bench discoveries and bedside applications requires not only a deep understanding of the underlying biology but also careful consideration of patient heterogeneity, comorbidities, and individual responsiveness. The success of HPC-based interventions ultimately depends on our ability to personalize hypoxic protocols based on each patient’s unique physiological profile, genetic background, and disease context. This personalized approach distinguishes modern HPC research from earlier, more empirical applications of hypoxic exposure.

#### 4.1.2. The Implementation of Hypoxia

More and more research about the implementation of HPC is being conducted. HPC in humans can be achieved through several methods, including high-altitude induced hypoxia, which was previously studied. However, due to its difficulty, researchers have since developed alternative devices [71]. Hypoxic chambers were invented to greatly mimic hypoxic environments, but they are too expensive to popularize; however, the devices could be repetitively used as methods in experimental animals.

Finally, we put emphasis on making devices to produce hypoxic gas as a traditional and mainstream way to induce intermittent hypoxia, based on which many advanced and practical devices are being invented. Using the devices, patients can inhale hypoxic gas mixtures (GHM) to induce HPC, and the method has become the mainstream way to induce HPC [72]. However, the selection of specific hypoxic strategies depends on several factors, which will be discussed below. Given the past research about hypoxic pathways, we realized that pharmacological preconditioning in pathways can also activate HPC [73]. We could also use chemical agents to mimic hypoxia [74]. Other ways like injecting hypoxic preconditioning cells cultured into animals are also reasonable [75]. Now, some probes are created to test hypoxic tolerance. They will give more information about patients before hypoxic training. Therefore, in the future, new and specific hypoxic patterns or doses still need to be discovered, including factors such as the degree, duration, frequency, and number of hypoxic episodes, considering the patient’s individual context, to provide safe and effective treatment.

Importantly, OSA-related intermittent hypoxia should not be equated with therapeutic hypoxic preconditioning. The latter is delivered in a controlled, limited, and non-lethal manner to induce adaptive protection. A table lists the differences between them (Table 3). For example, in our experimental paradigm, mice were exposed to 10 cycles/day of 5 min at 13% O_2_ alternating with 5 min at 21% O_2_ for 14 days. In contrast, OSA is a pathological sleep-related condition characterized by recurrent upper airway obstruction and repeated oxygen desaturation events, often occurring dozens of times per hour over several hours each night, thereby imposing a far greater cumulative hypoxic burden [76]. Moreover, OSA is accompanied by sleep fragmentation, sympathetic activation, oxidative stress, intrathoracic pressure swings, and systemic inflammation. These substantial differences in exposure pattern and physiological context explain why OSA does not constitute beneficial preconditioning [77].

### 4.2. The Frontier of Hypoxic Preconditioning Research: Immune Regulation and Anti-Tumor Effects

Based on current evidence, we believe that hypoxic preconditioning can regulate immune function and thereby influence various diseases, with effects similar to those of hypoxic environments [78,79]. HIF-1α influences various cellular metabolic and signaling pathways, such as glycolysis, oxidative phosphorylation, and the differentiation and function of immune cells. This further highlights the potential non-ischemic, direct targeting role of hypoxic preconditioning on cells (support cells, glial cells, immune cells). This includes the impact of HIF-1α on the Th17/Treg balance. Additionally, hypoxia influences both innate and adaptive immune responses by regulating IFN-α, TLR signaling pathways, and the metabolic state of immune cells [80,81,82]. In the central nervous system, microglia are key immune cells that determine the inflammatory microenvironment after ischemic or hypoxic injury. Excessive activation of pro-inflammatory microglia may aggravate neuronal damage, whereas anti-inflammatory or reparative microglia may promote tissue recovery. Scientists found that HPC shifted microglial responses toward a more protective phenotype after ischemic stroke, reduced the release of inflammatory cytokines, and enhanced neurovascular remodeling. Similarly, hypoxic preconditioning of mesenchymal stem cells has been reported to improve their survival and paracrine effects, which may contribute to tissue repair.

Hypoxia is considered a broad-spectrum regulator, and considerable attention has been directed toward exploring the potential effects of hypoxic preconditioning and different hypoxia combinations on various diseases. It is suspected that hypoxic conditioning and immunity are strongly correlated, prompting us to turn our attention to another disease closely related to immunity: cancer. The effect of hypoxia on the tumor microenvironment has long been recognized, and HIF-1α-related mechanisms have been extensively studied in cancer biology [83]. However, tumor-associated chronic hypoxia and therapeutic/adaptive hypoxic conditioning are not equivalent biological states. Chronic hypoxia within the tumor microenvironment is widely associated with malignant progression, whereas hypoxic preconditioning usually involves brief, controlled, intermittent exposure intended to induce adaptive responses. These distinct hypoxic patterns may activate different molecular and immunological pathways and may therefore produce different, or even opposite, effects on tumor biology [84,85]. In addition, the effects of hypoxic preconditioning may involve not only tumor cells themselves but also the tumor immune microenvironment.

Some studies have shown surprising effects, and this may represent a potential approach to cancer therapy. A 2006 study found intermittent hypoxia and hypoxia-mimetic agents can significantly prolong survival time with acute myeloid leukemia (AML) since they inhibit proliferation and promote differentiation of leukemic cells [86]. The differing results between in vivo and in vitro experiments suggest that the intervention target of hypoxic preconditioning may not only be the tumor cells themselves but also the tumor-induced microenvironment, including immune abnormalities. The differential effects of hypoxic preconditioning on the immune systems of hematological tumors and solid tumors suggest a possibility: immune cells are specifically sensitive to HIF-1α and hypoxia-related molecules [87].

Preclinical studies in animal tumor models suggest that appropriately designed intermittent hypoxia or hypoxic conditioning paradigms may exert antitumor effects under specific conditions. Meanwhile, a registered clinical study currently underway in China is exploring the potential effects of intermittent hypoxia on rectal cancer (NCT06584318), representing an important step in the clinical translation of hypoxic preconditioning research. This non-randomized, self-controlled clinical trial, which plans to enroll 20 colorectal cancer patients with recruitment scheduled to conclude by April, aims to evaluate the safety and efficacy of intermittent hypoxia—administered as periodic hypoxic–normoxic training designed to activate endogenous cellular protective mechanisms and enhance hypoxic adaptive responses—by comparing pre- and post-intervention outcomes within the same subjects, thereby translating the investigators’ prior murine evidence that short-term intermittent hypoxia bolsters immune function to suppress tumor progression into a preliminary human oncological setting. However, no studies have further investigated the underlying mechanisms, highlighting the need for a deeper understanding of hypoxic preconditioning, including the hypoxic model and preconditioning protocols.

Looking forward, the integration of HPC with other emerging therapeutic modalities holds tremendous promise. The combination of hypoxic preconditioning with immunotherapy, for instance, may enhance the efficacy of immune checkpoint inhibitors by remodeling the tumor microenvironment and reprogramming immune cell function [88,89]. Similarly, pairing HPC with regenerative medicine approaches, such as stem cell therapy or tissue engineering, could improve cell survival, engraftment, and functional integration [90,91]. The synergistic potential of these combinatorial strategies underscores the importance of viewing HPC not as a standalone therapy but as a versatile platform that can amplify the effects of multiple therapeutic interventions.

## 5. Conclusions

Hypoxic preconditioning lies at the intersection of evolutionary biology, molecular medicine, and clinical translation, illustrating how ancient adaptive mechanisms can be harnessed to address modern medical challenges. Hypoxia is a universal biological stress with context-dependent effects: severe or uncontrolled hypoxia causes tissue injury and disease progression, whereas mild, intermittent, and well-controlled hypoxic exposure can activate endogenous protective programs and enhance tolerance to subsequent severe hypoxic or ischemic insults. The protective effects of hypoxic preconditioning involve coordinated systemic, cellular, and molecular responses, including regulation of ventilation and circulation, metabolic remodeling, mitochondrial stabilization, calcium homeostasis, antioxidant defense, anti-inflammatory signaling, HIF-1α activation, adenosine signaling, ATP-sensitive potassium channels, neuroglobin, and other anti-hypoxia-related pathways. Recent advances in multi-omics, single-cell analysis, spatial profiling, and physiological monitoring have begun to reveal the complexity and heterogeneity of these adaptive responses. The next challenge is to integrate these findings into reliable mechanistic frameworks, identify clinically useful biomarkers, and establish individualized conditioning protocols with optimized intensity, duration, frequency, and timing. This is particularly important because the therapeutic window of hypoxic preconditioning may vary according to organ type, disease stage, age, and comorbidities. By transforming hypoxia from a harmful environmental stressor into a controllable therapeutic stimulus, hypoxic preconditioning may provide a simple, non-pharmacological, and potentially safe strategy for neuroprotection, cardiovascular protection, immune regulation, rehabilitation medicine, and selected anti-tumor applications. With further mechanistic validation and clinical optimization, “combating hypoxia with hypoxia” may become an important component of adaptive medicine and provide new opportunities for disease prevention and treatment.

## Figures and Tables

**Table 1:** Approximate oxygen availability and representative physiological effects across altitude ranges under standard atmospheric conditions.

Altitude (m)	Ambient PO_2_ (kPa)	Equivalent O_2_ Concentration (%)	Representative Physiological Effects
0–2000	21.2–16.6	20.9–16.4	Usually no obvious hypoxic symptoms; compensation generally not required
2000–4000	16.6–12.9	16.4–12.7	Compensatory respiratory and circulatory responses become evident
4000–7000	12.9–8.6	12.7–8.5	Incomplete compensation; marked physiological disturbance; impaired physical and cognitive performance
>7000	<8.6	<8.5	High risk of rapid loss of consciousness, muscle spasms, and other life-threatening events

**Note:** Values are approximate and based on standard atmospheric conditions. Equivalent O_2_ concentration refers to the normobaric oxygen percentage that would provide a similar ambient oxygen partial pressure at sea level.

**Table 2:** Representative molecular changes reported across repeated hypoxic preconditioning paradigms and their potential functional significance.

Factor	Full Name	Change	Main Functional Category	Biological Significance
LPO	Lipid peroxidation	Decreased	Oxidative stress	Reduced LPO indicates attenuation of oxidative membrane damage and improved cellular membrane stability.
ROS	Reactive oxygen species	Decreased	Oxidative stress	Lower ROS levels suggest reduced oxidative stress and less redox-mediated cellular injury.
SOD	Superoxide dismutase	Increased	Antioxidant defense	Increased SOD strengthens antioxidant capacity by scavenging superoxide radicals.
GSH-Px	Glutathione peroxidase	Increased	Antioxidant defense	Elevated GSH-Px enhances the removal of peroxides and protects against oxidative damage.
Intracellular Ca^2+^	Intracellular calcium ion	Decreased	Calcium homeostasis	Reduced intracellular Ca^2+^ helps prevent calcium overload, mitochondrial dysfunction, and cell injury.
Ca^2+^-ATPase	Calcium adenosine triphosphatase	Increased	Calcium homeostasis	Increased Ca^2+^-ATPase activity facilitates calcium extrusion or sequestration and helps maintain calcium balance.
Na^+^/K^+^-ATPase	Sodium-potassium adenosine triphosphatase	Increased	Ion homeostasis/energy metabolism	Increased Na^+^/K^+^-ATPase activity supports membrane potential maintenance and ionic homeostasis under hypoxic stress.
Lactate	Lactate	Decreased	Energy metabolism	Reduced lactate suggests improved metabolic efficiency and less reliance on anaerobic glycolysis.
FFA	Free fatty acids	Decreased	Energy metabolism	Lower FFA levels may reflect improved metabolic homeostasis and reduced lipotoxic stress.
TCA	Tricarboxylic acid cycle activity/intermediates	Increased	Energy metabolism	Increased TCA-related metabolism suggests improved aerobic energy production and mitochondrial adaptation.
HIF-1α	Hypoxia-inducible factor-1 alpha	Increased	Hypoxia signaling	Increased HIF-1α promotes transcriptional adaptation to hypoxia, including metabolic adjustment, angiogenesis, and cell survival pathways.
VEGF	Vascular endothelial growth factor	Increased	Angiogenesis/vascular adaptation	Elevated VEGF promotes angiogenesis and may improve tissue oxygen delivery.
Ngb	Neuroglobin	Increased	Oxygen handling/cytoprotection	Increased neuroglobin may improve oxygen utilization and enhance resistance to hypoxic injury.
Adenosine	Adenosine	Increased	Purinergic signaling/cytoprotection	Increased adenosine acts as an endogenous protective mediator that reduces energy demand and suppresses excitotoxicity.
ADORA1 Bmax	Adenosine A1 receptor maximum binding capacity	Decreased	Receptor regulation	Reduced ADORA1 Bmax may reflect adaptive receptor regulation after repeated conditioning.
ADORA1 affinity	Adenosine A1 receptor affinity	Increased	Receptor regulation	Increased ADORA1 affinity may enhance adenosine-mediated protective signaling.
Glutamate	Glutamate	Decreased	Excitatory neurotransmission	Lower glutamate reduces excitotoxicity, a major mechanism of hypoxic/ischemic neuronal injury.
Asp	Aspartate	Decreased	Excitatory neurotransmission	Reduced aspartate may also limit excitatory amino acid-mediated neurotoxicity.
GABA	Gamma-aminobutyric acid	Increased	Inhibitory neurotransmission	Increased GABA may reduce neuronal excitability and contribute to neuroprotection.
Gly	Glycine	Increased	Inhibitory neurotransmission/cytoprotection	Increased glycine may contribute to inhibitory regulation and membrane stabilization.
NE	Norepinephrine	Decreased	Neurohumoral regulation	Reduced NE may indicate attenuation of sympathetic activation and catecholamine-related stress.
DA	Dopamine	Increased	Neurotransmitter adaptation	Increased dopamine may reflect adaptive modulation of neurotransmission during hypoxic stress.
5-HT	5-Hydroxytryptamine	Increased	Neurotransmitter adaptation	Increased serotonin may participate in adaptive neuromodulatory responses to repeated hypoxia.
5-HIAA	5-Hydroxyindoleacetic acid	Increased	Monoamine metabolism	Increased 5-HIAA may reflect altered serotonin turnover during hypoxic preconditioning.
NPY	Neuropeptide Y	Decreased	Neuroendocrine/stress response	Reduced NPY may suggest attenuation of stress-related neuroendocrine and vasoconstrictive responses.
CGRP	Calcitonin gene-related peptide	Decreased	Neurovascular regulation	Decreased CGRP may reflect altered neurovascular and sensory peptide signaling during hypoxic preconditioning.
Ang II	Angiotensin II	Decreased	Vasoactive/hormonal signaling	Reduced Ang II may alleviate vasoconstriction, oxidative stress, and maladaptive cardiovascular responses.
PKC	Protein kinase C	Decreased	Signal transduction	Reduced PKC activity may indicate downregulation of stress-related signaling, although its effect is context-dependent.
PLA2	Phospholipase A2	Decreased	Membrane lipid signaling/inflammation	Reduced PLA2 may limit phospholipid breakdown and production of pro-inflammatory lipid mediators.
NOS	Nitric oxide synthase	Decreased	Nitric oxide signaling	Reduced NOS activity may lower nitrosative stress in some contexts, although the effect depends on the NOS isoform.
NO	Nitric oxide	Decreased	Nitric oxide signaling	Reduced excessive NO may help limit nitrosative injury, depending on the biological context.

**Note:** The molecular changes summarized here were reported across multiple repeated hypoxic preconditioning paradigms in the literature, including whole-body autohypoxia, low-pressure hypoxia, and intermittent hypoxia protocols. Because these responses were not all obtained under one standardized exposure condition, this table is intended as a representative integrative summary rather than a protocol-specific dataset. The direction and magnitude of these changes may vary depending on species, tissue type, hypoxic severity, exposure pattern, duration, and sampling time.

**Table 3:** Differences between therapeutic IHC and OSA-related intermittent hypoxia.

Feature	Therapeutic IHC	OSA-Related Intermittent Hypoxia
**Setting**	Controlled experimental/conditioning protocol	Pathological, sleep-related disorder
**Stimulus pattern**	10 cycles/day, once daily	Often dozens of events/hour during sleep
**Hypoxic phase**	5 or 10 min at 13% O_2_	Typically 10–30 s or longer per apnea/hypopnea event, with recurrent desaturation
**Reoxygenation**	5 min at 21% O_2_	Reoxygenation after airway reopening
**Frequency**	1 session/day	Recurrent throughout several hours of sleep each night
**Total burden**	Limited, predefined, non-injurious	High cumulative hypoxic burden
**Severity**	Moderate, controlled hypoxia	Variable but often deeper desaturation; SpO_2_ may fall to ~80% or lower in moderate-to-severe cases
**Biological consequence**	Protective conditioning	Cardiovascular complications

## Data Availability

No new data were created or analyzed in this study. Data sharing is not applicable to this article.
